# Thyroid Function and Anti-thyroid Antibodies in Pediatric Anti-NMDAR Encephalitis

**DOI:** 10.3389/fneur.2021.707046

**Published:** 2021-09-09

**Authors:** Lianfeng Chen, Wenlin Wu, Yang Tian, Yiru Zeng, Chi Hou, Haixia Zhu, Kelu Zheng, Yani Zhang, Yuanyuan Gao, Bingwei Peng, Sida Yang, Xiuying Wang, Shuyao Ning, Yinting Liao, Haisheng Lin, Kaili Shi, Xiaojing Li, Wen-Xiong Chen

**Affiliations:** Department of Neurology, Guangzhou Women and Children's Medical Center, Guangzhou Medical University, Guangzhou, China

**Keywords:** anti-NMDAR encephalitis, anti-thyroid antibody, thyroid hormone, non-thyroidal illness syndrome, children

## Abstract

**Objective:** Recent studies found that changes of thyroid antibodies (ATAbs), thyroid hormone, and non-thyroidal illness syndrome (NTIS) characterized by thyroid hormone inactivation with low triiodothyronine and high reverse triiodothyronine followed by suppressed thyroid-stimulating hormone (TSH) in adult anti-N-methyl-D-aspartate receptor (NMDAR) encephalitis were associated with disease severity. This study aimed to explore thyroid function and ATAbs in pediatric anti-NMDAR encephalitis and their clinical association.

**Methods:** We retrospectively analyzed the clinical data of 51 pediatric cases with anti-NMDAR encephalitis hospitalized in Guangzhou Women and Children's Medical Center from August 2016 to 2019.

**Results:** A percentage of 52.9% of patients belonged to the ATAb (+) group, with 26 cases both positive for anti-thyroid peroxidase antibodies (TPOAb) and anti-thyroglobulin antibodies (TGAb), and one patient only positive for TPOAb. A percentage of 62.7% of patients had at least one abnormality in terms of FT_3_, free thyroxin (FT_4_), or TSH levels. Meanwhile, 45.1% of patients were diagnosed with NTIS. Among 25 cases retested for thyroid function 2 months after the initial test, the respectively decreased FT_3_ and FT_4_ in 13 and 11 cases on admission returned to normal or closer normal than before; TPOAb in eight cases and TGAb in 12 cases were changed from positivity to negativity. Compared with onset, the level of TPOAb and TGAb at relapse remained stable or significantly decreased, respectively. Compared with the ATAb (–) group, the ATAb (+) group had an older onset age, a higher ratio of movement disorders, elevated rate of sleep disorders, increased anti-nuclear antibody positivity rate, and higher ratio of more than one course of intravenous immunoglobulin treatment. There were no significant differences between the NTIS and non-NTIS groups in clinical characteristics.

**Conclusion:** Anti-thyroid antibody positivity, abnormality of FT_3_, FT_4_, or TSH levels and NTIS are frequent in pediatric anti-NMDAR encephalitis. Thyroid antibody and thyroid hormone abnormalities could be improved through the course of treatment of anti-NMDAR encephalitis. Cases with ATAbs (+) are at older onset ages and more likely to be treated by intravenous immunoglobulin therapy more than once. Unlike adult anti-NMDAR encephalitis, NTIS might not be associated with the clinical characteristics of anti-NMDAR encephalitis in pediatric patients.

## Introduction

Anti-N-methyl-D-aspartate receptor (NMDAR) encephalitis is an autoimmune disorder associated with autoantibodies binding with the NR1 subunit of the NMDAR receptor (1). The common clinical manifestations of anti-NMDAR encephalitis include psychiatric symptoms, behavioral dysfunction, seizures, movement disorder, speech disorder, cognitive impairment, decreased consciousness, autonomic dysfunction, or central hypoventilation (2). Anti-NMDAR encephalitis can be accompanied with other autoantibodies such as the myelin oligodendrocyte glycoprotein antibody (3), similar to other immune-mediated diseases, presenting more than one immune disorder together (4). Thyroid antibodies are frequently detected not only in patients with autoimmune thyroid disease but also in individuals without overt thyroid dysfunction, including those with rheumatoid arthritis, type 1 diabetes mellitus, Crohn's disease, and neurological disorders such as multiple sclerosis (5). Thyroid hormones are essential in humans. During brain development, thyroid hormones play an important role in the proliferation and differentiation of neuronal and glial progenitors (6, 7). In addition, hypothyroidism is associated with a decreased hippocampus size, which is a major area involved in anti-NMDAR encephalitis (1, 8). Studies have found that thyroid hormones affect the prognosis of critical and severe diseases, besides substance metabolism, growth, and development (9). Recent studies have reported thyroid antibody (10) and hormone (11) changes in adult anti-NMDAR encephalitis associated with disease severity. Non-thyroidal illness syndrome (NTIS) is characterized by thyroid hormone inactivation, with low triiodothyronine and high reverse triiodothyronine, followed by suppressed thyroid-stimulating hormone (TSH); NTIS is associated with clinical characteristics of adult anti-NMDAR encephalitis (11). However, few such reports are addressing pediatric anti-NMDAR encephalitis. In this study, we aimed to evaluate thyroid function and anti-thyroid antibodies in pediatric anti-NMDAR encephalitis.

## Subjects and Methods

### Subjects

Children with anti-NMDAR encephalitis were retrospectively recruited from August 2016 to August 2019 in the Department of Neurology of Guangzhou Women and Children's Medical Center. This study was approved by the Ethics Committee of Guangzhou Women and Children's Medical Center. Written and signed consent was obtained from the patients' parents or guardians, who also explicitly consented to publish their personal details, clinical data, and images that could identify them.

### Methods

#### Inclusion Criteria

Patients aged younger than 18 years, diagnosed with anti-NMDAR encephalitis according to diagnostic criteria proposed by Graus et al. (2), and undergoing thyroid function tests including free triiodothyronine (FT_3_), free thyroxin (FT_4_), TSH, and anti-thyroid antibody (ATAb) tests including anti-thyroglobulin antibody (TGAb) and anti-thyroid peroxidase antibody (TPOAb) tests were involved.

#### Exclusion Criteria

Patients were excluded if they did not undergo thyroid hormone and thyroid function tests on admission, had known endocrine disorders or severe non-endocrine disorders that may influence thyroid function before anti-NMDAR encephalitis onset, or developed anti-NMDAR encephalitis after viral encephalitis.

#### Diagnostic Criteria of Hyperthyroidism, Subclinical Hyperthyroidism, and Non-thyroidal Illness Syndrome

Hyperthyroidism was diagnosed by endocrinologists according to the 2016 American Thyroid Association guidelines for diagnosis and management of hyperthyroidism (12). Subclinical hyperthyroidism was defined as a serum TSH concentration below the lower limit of the reference range, with serum FT_4_ and FT_3_ concentrations within their reference ranges. NTIS was characterized by the thyroid hormone inactivation with low triiodothyronine and high reverse triiodothyronine followed by suppressed TSH and was diagnosed based on serum FT_3_ below the age-appropriate normal level and low or normal TSH level (13–18).

#### Clinical Data Collection

Clinical data of the involved patients, including age at onset, gender, clinical manifestations, prodromal infection, laboratory test results, electroencephalogram (EEG), brain magnetic resonance imaging (MRI), treatments, outcome, and follow-up, were reviewed. The modified Rankin scale (mRS) was used to assess neurological disability at admission and discharge and at the end of follow-up (3, 19, 20). A poor response was defined as no mRS score improvement or mRS score ≥4 for 4 weeks (21). Relapse was defined as a new onset or worsening of symptoms occurring after at least 2 months of improvement or stabilization. The good long-term prognosis was defined as mRS score ≤ 2; the poor long-term prognosis was defined as mRS score >2 (21). Cerebrospinal fluid (CSF) pleocytosis was defined as a white blood cell (WBC) count > 15 × 10^6^/l in our center. The movement disorders in anti-NMDAR encephalitis include orofacial, limb, or trunk dyskinesias (2).

#### Laboratory Measurement

All included patients underwent thyroid function and ATAb examinations upon admission, which were repeated 2 months later in some individuals. Thyroid function indexes, including FT_3_, FT_4_, and TSH levels, and ATAbs (TGAb and TPOAb) were measured using highly sensitive magnetic antibody enzyme-linked immunoassays. The normal range for both TPOAb and TGAb was 0–60 IU/ml in terms of laboratory standards. TPOAb positivity was defined as a TPOAb level higher than 60 IU/ml. TGAb positivity was defined as a TGAb higher than 60 IU/ml. According to ATAb results, a patient with TPOAb or TGAb positivity was assigned to the ATAb-positive group; otherwise, the case was assigned to the ATAb-negative group. The normal ranges of FT_3_, FT_4_, and TSH levels in children vary with age.

NMDAR IgG in both serum and CSF samples was determined by cell-based assays (EUROIMMUN, Lübeck, Germany) and CSF glial fibrillary acidic protein IgG and serum myelin oligodendrocyte glycoprotein IgG. These methods have been reported in detail in our previous study (22).

#### Treatment

First-line immunotherapy was performed with intravenous methylprednisolone (IVMP) combined with intravenous immunoglobulin (IVIG) in the acute phase. Two weeks after the first course of IVIG, if patients were not improved well, patients would receive the second course of IVIG treatment or second course of IVMP treatment. Second-line treatment included rituximab or cyclophosphamide administration. In addition, one patient diagnosed with hyperthyroidism was treated with methimazole.

### Statistical Analysis

Statistical analysis was performed with IBM SPSS 20.0 for windows. Quantitative data with normal distribution were described as mean ± standard deviation and compared by Student's *t*-test or paired-sample *t*-test. Those with skewed distribution were presented as median with interquartile range (IQR) and compared by the Mann–Whitney *U*-test or Wilcoxon signed-rank test. Categorical data were described as frequency and percentage and compared by the chi-square test or Fisher's exact test. *p* < 0.05 (two-sided) was considered statistically significant.

## Results

### Demographic and Clinical Characteristic Data

A total of 51 patients (male: female, 21:30) were enrolled, and their clinical features have been reported in our previous study (23). Onset age was 6.3 ± 2.7 years, ranging from 1.9 to 15 years. Prodromal infections occurred in 12 cases (23.5%, 12/51), including respiratory infection (11 cases) and urinary tract infection (1 case). Major symptoms in the whole treatment course were psychiatric symptoms (88.2%, 45/51), seizures (84.3%, 43/51), speech disorders (80.4%, 41/51), sleep disorder (72.5%, 37/51), abnormal movement (68.6%, 35/51), fever (51.0%, 26/51), decreased consciousness (39.2%, 20/51), and autonomic dysfunction (5.9%, 3/51). The median initial mRS score evaluated on admission was 4 (IQR, 3–4). One patient diagnosed with hyperthyroidism had emotional lability, heat intolerance, bad temper, profuse sweating, weight loss, and increased appetite, accompanied by second-degree thyroid enlargement without pain on palpation.

### Ancillary Test Results

#### CSF Test

All patients underwent CSF examination after lumbar puncture in the acute phase. WBC count in the CSF at the first lumbar puncture was 44.4 ± 36.1 × 10^6^/l, and CSF pleocytosis was observed in 41.2% (21/51) of patients. Protein elevation in the CSF (reference value, 0.15–0.45 g/l) was only detected in 5.9% (3/51) of patients, with amounts that ranged from 0.53 to 2.09 g/l. The levels of glucose and chloride were normal except in one patient who showed decreased glucose (2.35 mmol/l; the ratio of CSF glucose to finger stick glucose was 2.35/4.00 = 0.59). In this patient with reduced CSF glucose, CSF WBC was 90 × 10^6^/l while the CSF protein level was within the normal reference range. These abnormalities of glucose level and WBC count in the CSF returned to normal after treatment with IVIG and IVMP, without antibiotic treatment. The CSF anti-NMDAR antibody was positive in all patients, while serum anti-NMDAR antibody was positive in 41.2% (21/51) of individuals.

#### ATAbs in Serum and Thyroid Function

On admission, all patients underwent examination of thyroid antibody tests, including serum TPOAb and TGAb. In total, five patients (9.8%, 5/51) received IVIG in other hospitals before thyroid antibody tests in our center. Among them, three patients received two courses of IVIG treatment, and two patients received one course of IVIG treatment. The median level of TPOAb and TGAb was 249.9 IU/ml (IQR, 48.0–454.7 IU/ml) and 78.6 IU/ml (IQR, 15.9–319.8 IU/ml), respectively. Twenty-seven cases (52.9%, 27/51) are TPOAb positive, and their median level of TPOAb was 373.7 IU/ml (IQR, 246.7–612.6 IU/ml). Twenty-six cases (51.0%, 26/51) are TGAb positive, of which the median level of 319.8 IU/ml (IQR, 183.8–499.4 IU/ml) and their TPOAb were positive as well. In total, 52.9% (27/51) of patients belonged to the ATAb (+) group, and 47.1% (24/51) were from the ATAb (–) group.

On admission, all included patients underwent examinations for serum FT_3_, FT_4_, and TSH. The median levels of FT_3_, FT_4_, and TSH were 4.91 pmol/l (IQR, 4.04–5.71 pmol/l), 18.78 pmol/l (IQR, 16.93–20.40 pmol/l), and 1.420 μIU/ml (IQR, 0.841–2.344 μIU/ml), respectively. Totally, 62.7% (32/51) of patients had at least one abnormality of FT_3_, FT_4_, or TSH level compared with age-appropriate normal levels. Abnormal FT_3_ was seen in 47.1% (24/51) of patients, including FT_3_ reduction (95.8%, 23/24) and FT_3_ elevation (4.2%, 1/24). Abnormal FT_4_ was found in 45.1% (23/51) of patients, including FT_4_ decrease (82.6%, 19/23) and FT_4_ elevation (17.4%, 4/23). Decreased TSH as an abnormality was observed in 39.2% (20/51) of patients. Combining FT_3_, FT_4_, and TSH to classify these abnormalities (more details in Table 1), patients with concurrently decreased FT_3_, FT_4_, and TSH (40.6%, 13/32) were the most frequent group, followed by those with decreases in both FT_3_ and FT_4_ but normal TSH (12.5%, 4/32). Decreased TSH and normal FT_3_ and FT_4_ levels were only found in three patients (9.4%, 3/32) who did not show the clinical presentations of hyperthyroidism. However, the thyroid function test in these three patients showed that TSH levels returned to normal at 2 months after the first test; therefore, these three patients did not meet the criteria for diagnosing subclinical hyperthyroidism. Increased FT_3_ and FT_4_ amounts and decreased TSH levels were only detected in one patient diagnosed with hyperthyroidism. According to the NTIS diagnosis criteria, 45.1% (23/51) of patients were diagnosed with NTIS, while 54.9% (28/51) did not have NTIS. The 28 patients with no NTIS included 19 individuals with normal FT_3_, FT_4_, and TSH levels, three cases with decreased TSH and normal FT_3_ and FT_4_ levels, three cases with increased FT_4_ and normal FT_3_ and TSH levels, two cases with decreased FT_4_ and normal FT_3_ and TSH levels, and the remaining one patient diagnosed with hyperthyroidism.

**Table 1:** Results of thyroid hormone test on admission.

**TSH/FT_**3**_/FT_**4**_**	**TSH (median, IQR,** **or mean ± SD,** **μIU/ml)**	**FT_**3**_ (median, IQR,** **or mean ± SD,** **pmol/l)**	**FT_**4**_ (median, IQR,** **or mean ± SD,** **pmol/L)**	**Number of patients** **(*n*1, %^a^)**	**ATAb (+)** **(*n*2, %^b^)**	**ATAb (–)** **(*n*3, %^c^)**
NNN	2.116 (1.098, 2.885)	5.14 (4.84, 5.71)	19.1 (18.27, 20.18)	19 (37.3%)	9 (47.4%)	10 (52.6%)
NN↑	2.119 ± 0.829	5.57 ± 1.15	24.23 ± 0.88	3 (5.9%)	2 (66.7%)	1 (33.3%)
NN↓	1.261 ± 0.490	5.36 ± 0.42	14.37 ± 1.29	2 (3.9%)	1 (50.0%)	1 (50.0%)
N↓N	1.862 ± 0.651	5.35 ± 1.72	18.27 ± 0.53	3 (5.9%)	0 (0.0%)	3 (100.0%)
N↓↓	1.673 (1.286, 2.312)	3.29 (3.23, 3.44)	12.72 (11.96, 13.51)	4 (7.8%)	3 (75.0%)	1 (25.0%)
↓*↓↓*	1.219 (0.687, 2.055)	4.73 (3.38, 5.87)	18.58 (13.06, 20.46)	13 (25.5%)	9 (69.2%)	4 (30.8%)
↓↓N	0.450 ± 0.203	3.78 ± 0.26	17.20 ± 1.71	3 (5.9%)	2 (66.7%)	1 (33.3%)
↓NN	0.251 ± 0.125	5.38 ± 1.06	22.70 ± 3.53	3 (5.9%)	0 (0.0%)	3 (100.0%)
↓*↑↑*	0.001	94.3	>30.8	1 (2.0%)	1 (100.0%)	0 (0.0%)
Total	1.420 (0.841, 2.344)	4.91 (4.05, 5.71)	18.78 (16.93–20.40)	51 (100%)	27 (52.9%)	24 (47.1%)

*N, normal; ↓, decrease; ↑, increase*;

a *n1/51 × 100%*;

b *n2/n1 × 100%*;

c *n3/n1 × 100%; FT_3_, free triiodothyronine; FT_4_, free thyroxin; TSH, thyroid-stimulating hormone*.

Two months after the first test, 49.0% (25/51) of patients were reexamined for thyroid function and ATAbs, including 13 out of 23 cases with initially decreased FT_3_, 11 out of 19 cases with decreased FT_4_, 18 out of 27 cases with TPOAb positivity, and 17 out of 26 cases with TGAb positivity. The results showed that FT_3_ levels in all 13 patients with initially decreased FT_3_ significantly returned to the normal range or maintained higher than before (6.1 ± 1.9 pmol/l vs. 4.7 ± 1.4 pmol/l; Mann–Whitney *U*-test, *Z* = −2.866, *p* < 0.01); FT_4_ levels in all 11 patients with initially decreased FT_4_ were also significantly increased to the normal range (21.5 ± 5.3 pmol/l vs. 18.1 ± 4.4 pmol/l; Mann–Whitney *U*-test, *Z* = −2.597, *p* < 0.01). TPOAb amounts in all 18 patients with initial TPOAb positivity significantly decreased from 446.2 IU/ml (IQR, 243.6–670.1 IU/ml) to 85.3 IU/ml (IQR, 42.9–225 IU/ml) (Mann–Whitney *U*-test, *Z* = −3.101, *p* < 0.01); TPOAb levels in eight of these 18 patients (44.4%, 8/18) became negative. TGAb amounts in the 17 patients with initial TGAb positivity significantly decreased from 211.5 IU/ml (IQR, 146.9–337.3 IU/ml) to 24.9 IU/ml (IQR, 17.3–73.5 IU/ml) (Mann–Whitney *U*-test, *Z* = −2.343, *p* = 0.02); TGAb levels in 12 of these 17 patients (70.6%, 12/17) became negative. Only one patient, diagnosed with hyperthyroidism, had repeated evaluation of ATAbs and thyroid function 4 months after treatment with methimazole, and the results showed that the increased levels of TSH from 0.001 to 0.023 μIU/ml, decreased levels of FT_3_ from >30.8 to 7.44 pmol/l, normalized levels of FT_4_ from 94.3 pmol/l, and decreased levels of TGAb from >500 to 393.2 IU/ml were revealed; TPOAb levels both on admission and reexamination were >1,300 IU/ml.

#### Serum Pathogenic Test

All patients underwent pathogenic serum tests including IgM antibodies for human Chlamydia psittaci, Mycoplasma pneumonia, Legionella pneumonia type 1, Coxiella burnetii, respiratory adenovirus, influenza A, influenza B, parainfluenza, respiratory syncytial virus, and herpes simplex virus. The results showed positive outcomes in the 13 cases (25.5%, 13/51), including eight cases that are serum Herpes simplex virus IgM positive and five cases that are mycoplasma pneumonia IgM positive, respectively.

#### Other Coexisting Autoantibodies in Serum and CSF

Totally, 51 patients underwent a coexisting autoantibody test for serum and CSF samples. Except for anti-NMDAR antibody and ATAbs, the other autoantibodies tested included IgG antibodies for glutamate and γ-aminobutyric acid alpha and beta receptors, alpha-amino-3-hydroxy-5-methyl-4-isoxazolepropionic acid receptor1, alpha-amino-3-hydroxy-5-methyl-4-isoxazolepropionic acid receptor2, leucine-rich glioma-inactivated 1 protein, and contactin-associated protein-like 2 in CSF and serum, IgG antibodies for myelin oligodendrocyte glycoprotein and aquaporin-4 in serum, and antinuclear antibody, anti-nuclear ribonucleoprotein antibody, anti-Sjogren syndrome-related antigen A (SSA)/52 KD, SSA/60 KD, and anti-Sjogren syndrome-related antigen B in serum. A percentage of 21.6% (11/51) of patients showed positivity for other autoantibodies. Only anti-nuclear antibody (ANA) positivity was seen in seven cases (13.7%, 7/51), and only SSA positivity in two cases (3.9%, 2/51); both ANA and SSA positivities were detected in 2 cases (3.9%, 2/51). Myelin oligodendrocyte glycoprotein antibody positivity was seen in one patient.

#### EEG

All patients underwent EEG. Totally 49 cases had abnormal EEG, including 88.2% (45/51) with slow wave, 35.3% (18/51) with epileptic activity, and 2.0% (1/51) with Delta brush.

#### Imaging Examination

All patients underwent brain MRI examination in the acute phase, and 15.7% (8/51) had parenchymal lesions, with the most common location being the temporal lobe (five cases), followed by the insular lobe (four cases). Totally three patients underwent ultrasound examination of the thyroid: two cases had a normal organ, while a patient diagnosed with hyperthyroidism showed diffuse goiter and thyroid inferno. All patients underwent tumor screening, including chest computer tomography scans and abdominal and genital MRI scans. No patients had tumors.

### Treatment

All patients were treated by first-line immunotherapy with IVMP combination with IVIG. Thirty-two patients received two courses of IVIG treatment, and 19 patients received only one course of IVIG treatment. Forty-nine patients received one course of IVMP treatment, and only two patients received two courses of IVMP treatment. Totally, 78.4% (40/51) of patients had a good response to the first-line therapy. The remaining 11 patients had poor response to the first-line immunotherapy, among whom six cases were subsequently treated with rituximab as second-line immunotherapy and improved without relapse; five patients continued treatment with IVIG monthly (a total of 400 mg/kg/day monthly) and a low oral dose of corticosteroid treatment (0.5–1 mg/kg/day of prednisone for 1–2 weeks, with dosage tapering weekly for 3–6 months). Totally, three out of these five patients, who continued to be treated with IVIG and a low oral dose of corticosteroid, improved without relapse, while the remaining two cases relapsed and were further treated with cyclophosphamide. Consequently, after treatment with cyclophosphamide, these two patients improved without relapse.

In addition, 15 cases were treated with antiepileptic drugs—of these, nine and six patients with two types of antiepileptic drugs for seizures' control needed.

One patient diagnosed with hyperthyroidism was treated with methimazole and a low-iodine diet.

As supportive treatment, one patient received tracheal intubation and mechanical ventilation. Totally three cases accompanied by infection received antibiotic therapy, including meropenem (two cases) and a combination of vancomycin and cefoperazone-sulbactam (one case). In addition, nine cases were treated with risperidone to improve the abnormal mental behaviors.

### Course and Relapse

The median length of the first hospital stay was 28.8 ± 10.2 days. Seven patients (13.7%, 7/51) relapsed during corticosteroid weaning (three cases) or after withdrawal (four cases). Compared with TPOAb levels at onset, TPOAb amounts on relapse tended to decrease without statistical significance [341.8 IU/ml (IQR, 110.6–902.4 IU/ml) at onset vs. 34.6 IU/ml (IQR, 30.2–145.9 IU/ml) at relapse; Wilcoxon signed-rank test, *p* = 0.07]. In addition, compared with TGAb amounts at onset, TGAb levels on relapse were significantly decreased [295.8 IU/ml (IQR, 78.2–482.4 IU/ml) at onset vs. 16.6 IU/ml (IQR, 15–23.45 IU/ml) at relapse; paired-sample *t*-test, *p* = 0.02].

### Prognosis

One patient (2.0%) was lost to follow-up for poor compliance. The duration of follow-up was 20.0 ± 6.7 months. The median initial mRS score in the 50 followed patients was 4 (IQR, 3–4), which was significantly higher than the value at the last follow-up of 0 (IQR, 0–1) (*Z* = −6.386, Wilcoxon signed-rank test, *p* < 0.01). All the followed-up patients were alive at the last follow-up and achieved a good prognosis (mRS ≤ 2).

### Clinical Comparison Between ATAb (+) and ATAb (–) Groups

There was no significant difference in gender distribution between the ATAb (+) and ATAb (–) groups, whereas onset age was elevated in the ATAb (+) group compared with the ATAb (–) group (7.1 ± 2.9 vs. 5.3 ± 2.2 years, *p* = 0.02). As for clinical characteristics, compared with the ATAb (–) group, the ATAb (+) group had higher rates of movement disorders (88.9 vs. 45.8%, *p* < 0.01) and sleep disorders (85.2 vs. 58.3%, *p* = 0.03), ANA positivity (29.6 vs. 4.1%, *p* = 0.03), and IVIG treatment ≥2 courses (77.8 vs. 45.8%, *p* = 0.02). Details are shown in Table 2.

**Table 2:** Comparison of clinical characteristics between the ATAb (+) and ATAb (–) groups.

	**ATAbs (+)**	**ATAbs (–)**	***p***
	***n* = 27**	***n* = 24**	
Male/female	12/15	9/15	0.62^a^
Age (mean ± SD,years)	7.1 ± 2.9	5.3 ± 2.2	0.02^b^
Fever, *n* (%)	15 (55.6)	11 (45.8)	0.49^a^
Decrease consciousness, *n* (%)	13 (48.1)	7 (29.1)	0.49^a^
Movement disorders, *n* (%)	24 (88.9)	11 (45.8)	<0.01^a^
Epileptic seizures, *n* (%)	22 (81.4)	21 (87.5)	0.71^c^
Speech disorders, *n* (%)	24 (88.9)	17 (70.8)	0.16^c^
Psychiatric symptoms, *n* (%)	26 (96.3)	19 (79.1)	0.09^c^
Sleep disorders, *n* (%)	23 (85.2)	14 (58.3)	0.03^a^
Prodromal infections, *n* (%)	9 (33.3)	3 (12.5)	0.08^a^
HSV IgM (+) *n* (%)	4 (14.8)	4 (16.7)	1.00^c^
Pleocytosis in CSF, *n* (%)	12 (44.4)	9 (37.5)	0.62^a^
ANA positive, *n* (%)	8 (29.6)	1 (4.1)	0.03^c^
Lesions in brain MRI, *n* (%)	4 (14.8)	4 (16.7)	1.00^c^
IVIG (≥2 courses), *n* (%)	21 (77.8)	11 (45.8)	0.02^a^
Rituximab, *n* (%)	4 (14.8)	2 (8.3)	0.67^c^
Hospital day (d)	31.0 ± 9.9	26.1 ± 9.6	0.06^d^
mRS on admission	4 (3, 4)	4 (3, 4)	0.40^d^
mRS at discharge	2 (2, 3)	2 (2, 3)	0.55^d^
Relapse, *n* (%)	5 (18.5)	2 (8.3)	0.43^c^

a *Chi-square test*;

b *independent t-test*;

c *Fisher's exact test*;

d *Mann–Whitney U-test*.

*ANA, antinuclear antibody; CSF, cerebrospinal fluid; HSV, Herpes simplex virus; IVIG, intravenous immunoglobulin; MRI, magnetic resonance imaging; mRS, modified Rankin scale; SD, standard deviation*.

### Clinical Comparison Between Patients With and Without NTIS

Patients with anti-NMDAR encephalitis were divided into the NTIS and non-NTIS groups based on serum FT_3_ and TSH amounts. There were no significant differences between these two groups in gender distribution, onset age, clinical symptoms, and rates of prodromal infections, pleocytosis in CSF, ANA positivity, lesions in brain MRI, IVIG treatment ≥2 courses and treatment with rituximab, and mRS score on admission or at first discharge and relapse rate (more details in Table 3).

**Table 3:** Comparison of clinical characteristics between NTIS and not NTIS group.

	**NTIS**	**Not NTIS**	***p***
	***n* = 23**	***n* = 28**	
Male/female	9/14	12/16	0.79^a^
Age (mean ± SD, years)	6.5 ± 2.7	6.0 ± 2.7	0.66^b^
Fever, *n* (%)	9 (39.1)	17 (60.7)	0.13^a^
Decrease consciousness, *n* (%)	10 (43.5)	10 (35.7)	0.57^a^
Movement disorders, *n* (%)	15 (65.2)	20 (71.4)	0.63^a^
Epileptic seizures, *n* (%)	18 (78.3)	25 (89.3)	0.28^c^
Speech disorders, *n* (%)	18 (78.3)	23 (82.1)	0.74^c^
Psychiatric symptoms, *n* (%)	21 (91.3)	24 (85.7)	0.68^c^
Sleep disorders, *n* (%)	16 (69.6)	21 (75.0)	0.67^a^
Prodromal infections, *n* (%)	6 (26.1)	6 (21.4)	0.70^a^
Pleocytosis in CSF, *n* (%)	7 (30.4)	14 (50.0)	0.16^c^
ANA positive, *n* (%)	3 (13.0)	6 (21.4)	0.48^c^
Lesions in brain MRI, *n* (%)	6 (26.1)	2 (7.1)	0.12^c^
IVIG (≥2 courses), *n* (%)	15 (65.2)	17 (60.7)	0.95^a^
Rituximab, *n* (%)	3 (13.0)	3 (10.7)	1.00^c^
Hospital day (d)	27.3 ± 10.2	29.8 ± 9.8	0.32^d^
mRS on admission	4 (3.4)	4 (4.4)	0.92 ^d^
mRS at discharge	3 (2.4)	2 (1.3)	0.10^d^
Relapse, *n* (%)	2 (8.7)	5 (17.9)	0.44^c^

a *Chi-square test*;

b *independent t-test*;

c *Fisher's exact test*;

d *Mann–Whitney U-test*.

*ANA, antinuclear antibody; CSF, cerebrospinal fluid; IVIG, intravenous immunoglobulin; MRI, magnetic resonance imaging; mRS, modified Rankin Scale; SD, standard deviation*.

## Discussion

In the present study, we aimed to evaluate thyroid function and ATAbs in pediatric anti-NMDAR encephalitis. We found that 52.9% of patients had TPOAb positivity, and 51.0% had TGAb positivity. Totally 52.9% of patients belonged to the ATAb (+) group. ATAbs also occur in immune-mediated nervous diseases such as multiple sclerosis (24), optic neuromyelitis (25, 26), limbic encephalitis (27), Hashimoto's encephalopathy (28), and adult anti-NMDAR encephalitis (10). ATAbs in adult anti-NMDAR encephalitis were reported in 52.4% of cases, corroborating the current findings (10). The prevalence rates of ATAbs in a study of adult anti-NMDAR encephalitis and ours were distinctly higher than those of 10–15% found in the general population (27, 28). Besides, we also found that patients in the ATAb (+) group had partly different characteristics than the ATAb (–) group. The ATAb (+) group at older onset age had a higher rate of movement and sleep disorders, had elevated frequencies of ANA positivity, needed more courses of IVIG treatment, and had longer hospital stays. Although in the previous report, the adult patients with anti-NMDAR encephalitis in the ATAb (+) group also showed different clinical characteristics from the ATAb (–) group, and those differences were not the same findings in the current work (10). Specifically, the adult patients with anti-NMDAR encephalitis in the ATAb (+) group had a higher mRS score at admission or discharge, a higher rate of epileptic seizures and consciousness disorder, and an increased rate of lesions in brain MRI. However, the adult study did not compare the difference in the rates of movement disorders and sleep disorders, ANA positivity, and the course of IVIG treatment between the ATAb (+) and ATAb (–) groups (10). In addition, we also found no differences in the rates of seizures, conscious disorder, and lesions in brain MRI between the ATAb (+) and ATAb (–) groups. The difference between this trial and the adult study might be caused by the difference of study subjects or the limited sample size. Although there were differences between the ATAb (+) and ATAb (–) groups, it is unclear whether ATAbs play a direct pathogenic role in anti-NMDAR encephalitis. It was reported that thyroid globulin might cross-react with myelin-related antigen epitopes in molecular simulation, indicating that TGAb could cause brain injury (24). In addition, TPOAb could specifically bind to cerebellar astrocytes in patients with Hashimoto's encephalopathy (29). It was speculated that TPOAb might affect glial function after interacting with glial cells, thus resulting in neuronal dysfunction (29). However, in this study, ATAbs were not associated with disease severity at onset and the level of TGAb decreased at relapse, and TPOAb remained stable at relapse. We speculated that elevated ATAbs might be associated with increased hyperactivity immune response at the onset of anti-NMDAR encephalitis in the ATAb (+) group, and these patients required more courses of IVIG treatment. However, anti-NMDAR encephalitis relapse was not associated with elevated ATAbs in this study.

We found that 62.7% of patients had at least one abnormality of FT_3_, FT_4_, or TSH levels compared with age-appropriate normal values. Abnormalities of FT_3_, FT_4_, and TSH were found in 47.1%, 45.1%, and 39.2% of cases, respectively. In addition, 45.1% (23/51) of patients were diagnosed with NTIS, also known as low triiodothyronine syndrome, and could be caused by some pathologies in the absence of thyroid disease (30). NTIS occurs in many hospitalized patients, especially critically ill patients in the ICU (31, 32). NTIS has also been reported in neuroimmune diseases, including anti-NMDAR encephalitis (11, 24–26). Studies showed that adult patients with anti-NMDAR encephalitis with NTIS have a higher rate of decreased consciousness, an elevated mRS score at admission, and longer hospital stay (11). However, in this study, no significant differences were found in clinical symptoms, mRS scores at admission or discharge, and hospital stay between the NTIS and non-NTIS groups. The inconsistency between the adult trial and this study may be caused by differences in study subjects. In addition, we could not fully confirm that NTIS had no effects on pediatric anti-NMDAR encephalitis due to the small sample size and only a single time point at admission for the thyroid function tests. NTIS could occur later after hospital admission, so some possible NTIS cases might not have been detected, which might affect the finding that NTIS was not associated with clinical features. Further investigation with large sample size and multiple time point tests for the thyroid function tests is required.

In NTIS, thyroid hormone treatment is controversial. Some researchers believed that proper thyroid hormone therapy is beneficial to disease rehabilitation (30). However, the benefit of thyroid hormone treatment is not always achieved (33, 34). A double-blind randomized placebo-controlled study of children with NTIS after cardiac surgery found that thyroid hormone treatment failed to improve growth and neurodevelopment after long-term observation (33). Meanwhile, others disagreed with thyroid hormone treatment in NTIS patients and considered that NTIS compensatorily responded to diseases and aggressive treatment might be counterproductive (9). The present study did not perform thyroid hormone treatment for NTIS. Nevertheless, FT_3_, FT_4_, or TSH levels returned to normal or significantly increased, accompanied by improved anti-NMDAR encephalitis. Actually, NTIS may be related to variations of inflammatory factors (30). After primary disease improvement, inflammation in the primary disease could be controlled, and NTIS would also be improved. Therefore, we hypothesized that NTIS in pediatric anti-NMDAR encephalitis is a temporary change in an acute phase of the disease, which requires follow-up and can recover without thyroid hormone treatment.

Our study has several limitations. The first limitation is that only a single time point at admission for the thyroid function tests may affect the finding that NTIS was not associated with clinical features. The second limitation is that our study is a retrospective study and lacks follow-up thyroid antibody and thyroid function testing in around half of the subjects. In addition, in terms of the ANA antibody test, ANA positivity was only seen in a total of nine cases, among whom only two patients underwent the concentration of the ANA antibody test (33.32 and 90.29 IU/l, respectively). For the limitation of the retrospectively study, we could not provide the ANA values for the other seven patients.

## Conclusion

Anti-thyroid antibody positivity, abnormality of FT_3_, FT_4_, or TSH levels and NTIS are frequent in pediatric anti-NMDAR encephalitis. Thyroid antibody and thyroid hormone abnormalities could be improved through the course of treatment of anti-NMDAR encephalitis. Cases with ATAbs (+) at older onset ages are more likely to be treated by intravenous immunoglobulin therapy more than once. Unlike adult anti-NMDAR encephalitis, NTIS might not be associated with the clinical characteristics of anti-NMDAR encephalitis in pediatric patients.

## Data Availability Statement

The raw data supporting the conclusions of this article will be made available by the authors, without undue reservation.

## Ethics Statement

This study was approved by the Ethics Committee of Guangzhou Women and Children's Medical Center (2019052419364384). Written informed consent to participate in this study was provided by the participants' legal guardian/next of kin.

## Author Contributions

LC, WW, YT, and YiZ study concept, acquisition of data, and drafting of the manuscript. CH, HZ, KZ, YaZ, and YG study concept and acquisition of data. BP, SY, XW, SN, YL, HL, and KS analysis of data and interpretation. XL and W-XC study concept and critical revision of the manuscript for intellectual content. All the authors gave final approval of the version to be published.

## Funding

This present study was supported by the Health and Family Planning Technological Project Foundation of Guangzhou City (Grant No. 20181A011038). The funders had no role in the study concept, study design, data analysis, interpretation, or reporting of the results. The authors had full control of the data and information submitted for publication.

## Conflict of Interest

The authors declare that the research was conducted in the absence of any commercial or financial relationships that could be construed as a potential conflict of interest.

## Publisher's Note

All claims expressed in this article are solely those of the authors and do not necessarily represent those of their affiliated organizations, or those of the publisher, the editors and the reviewers. Any product that may be evaluated in this article, or claim that may be made by its manufacturer, is not guaranteed or endorsed by the publisher.

## Abbreviations

ATAbs: thyroid antibodies
NMDAR: N-methyl-D-aspartate receptor
ANA: anti-nuclear antibody
CSF: cerebrospinal fluid
EEG: electroencephalograph
FT_3_: free triiodothyronine
FT_4_: free thyroxin
IQR: interquartile range
IVIG: intravenous immunoglobulin
IVMP: intravenous methylprednisolone
MRI: brain magnetic resonance imaging
mRS: modified Rankin scale
NTIS: non-thyroidal illness syndrome
SSA: anti-Sjogren syndrome-related antigen A
TGAb: anti-thyroglobulin antibodies
TPOAb: anti-thyroid peroxidase antibodies
TSH: thyroid-stimulating hormone.
